# A Case Series on Cardiac and Skeletal Involvement in Two Families with PRKAG2 Mutations

**DOI:** 10.1155/2019/7640140

**Published:** 2019-03-26

**Authors:** Anita Sri, Piers Daubeney, Sanjay Prasad, John Baksi, Maria Kinali, Inga Voges

**Affiliations:** ^1^Imperial College London, South Kensington Campus, London SW7 2AZ, UK; ^2^Royal Brompton and Harefield NHS Foundation Trust, Sydney Street, London SW3 6NP, UK; ^3^Department of Congenital Heart Disease and Paediatric Cardiology, University Hospital Schleswig-Holstein, Campus Kiel, Kiel, Germany

## Abstract

**Background:**

PRKAG2 is a rare autosomal dominant syndrome that mainly presents with hypertrophic cardiomyopathy, ventricular preexcitation, and conduction abnormalities. This case report demonstrates that the PRKAG2 mutation presents with various phenotypes already in pediatric patients.

**Case Summary:**

We describe the clinical and investigative findings in two families with a PRKAG2 mutation from the different variants in the gene on chromosome 7q36.1, emphasising that the variability of phenotypes and that presentation in childhood is common. Furthermore, we highlight that skeletal myopathy and hypertrophic cardiomyopathy are significant debilitating characteristics of the PRKAG2 mutation.

**Conclusion:**

In our report of adult and pediatric patients, early presentation in childhood with hypertrophic cardiomyopathy and skeletal muscle involvement was common, demonstrating the challenges of the clinical management of PRKAG2 mutations.

## 1. Introduction

PRKAG2 is an autosomal dominant syndrome associated with left ventricular hypertrophy, ventricular preexcitation, and progressive conduction abnormalities [1]. This disorder has been described as rare with a prevalence of 0.23–1% in patients with hypertrophic cardiomyopathy [1]. Affected individuals often present at a young adult age but manifestation in childhood has been described [2, 3]. The spectrum of symptoms is broad and can include signs of skeletal muscle involvement [4].

This case series of two families with PRKAG2 mutation describes the clinical and investigative findings in pediatric and adult patients and highlights the variability of phenotypes.

Two families, family A and family B, consist of 8 carrying PRKAG2 variants (5 and 3 individuals, respectively) were managed in the same tertiary centre.

## 2. Family A

Family A, of white Caucasian origin, has the c.1463A > T (p.Asn488lle) mutation confirmed in 3 generations with 4 members diagnosed (Table 1). Electrocardiogram and echocardiographic findings are listed in Table 2.

The proband in Family A is the grandfather, first in the family to be diagnosed with PRKAG2 gene mutation, who was managed at another tertiary centre. He presented with evidence of skeletal myopathy and underwent dual-chamber implantable cardioverter defibrillator implantation.

**Figure 1:**
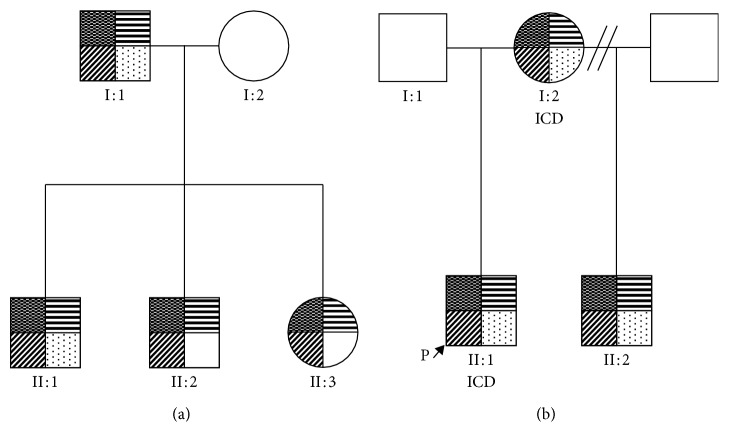
A pedigree of two families with PRKAG2 variant mutations and phenotypes. Upper left quadrant shaded indicated confirmed PRKAG2-positive mutation. Upper right quadrant shaded indicated hypertrophic cardiomyopathy. Lower left quadrant shaded indicated preexcitation identified on ECG, and lower right quadrant shaded indicated skeletal myopathy. ICD, implantable cardiac defibrillator.

**Figure 2:**
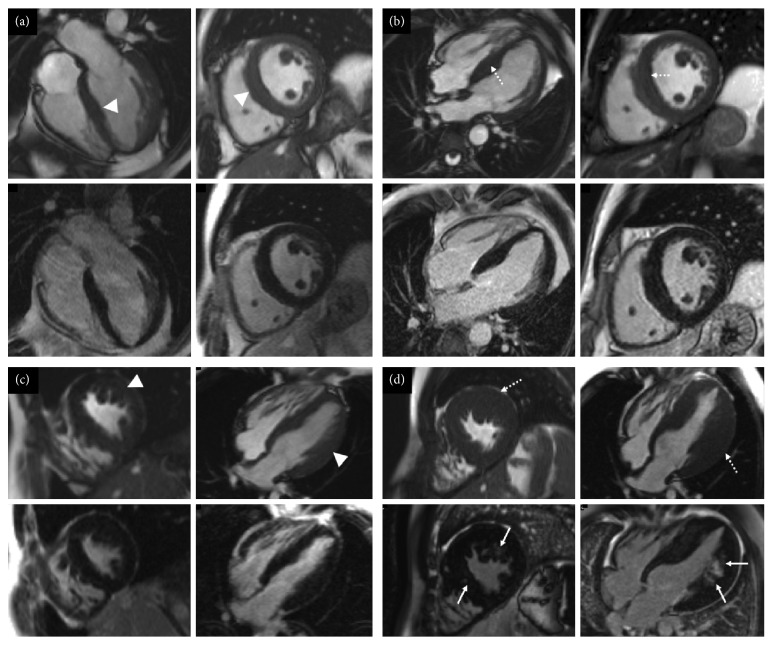
Short-axis and four chamber cine images from the initial and latest cardiovascular magnetic resonance scans of I:1 (a, b) and (c, d) II:1 from family A. Top row for each set of four images shows cine images, and the bottom row shows late gadolinium enhancement images. (a) On the first study, there is increased left ventricular wall thickness (maximum 12 mm, white arrowheads). No late gadolinium enhancement is present. (b) The latest scan shows marked asymmetric left ventricular hypertrophy (maximum 22 mm, dotted arrows) with patchy myocardial fibrosis (white arrows). (c) The first cardiovascular magnetic resonance scan showed moderate asymmetric septal hypertrophy (maximum 16 mm, white arrowheads) which appears to have mildly increased on the latest scan ((d), white arrows). (c, d) No areas of late gadolinium enhancement are identified.

The proband's son (I:1 in Figure 1(a)), now a 38-year-old gentleman, was diagnosed during family screening. He first presented with reduced exercise tolerance but no other cardiovascular symptoms. His initial electrocardiogram showed a short PR interval with left bundle branch block, and during follow-up, delta waves emerged. Echocardiogram and cardiovascular magnetic resonance at initial presentation showed asymmetric septal hypertrophy with a maximum wall thickness of 20 mm (Figures 2(a) and 2(b)) and no evidence of infiltrative fibrosis. Four years later, he complained of waking up during the night several days in a week because of leg pains and struggled with activities of daily living.

The proband's eldest grandson from I:1 (II:1 in Figure 1(a)), is a 15-year-old male, referred to the cardiomyopathy team, at 11 years old for screening. He was diagnosed with hypertrophic cardiomyopathy and skeletal myopathy. His initial electrocardiogram showed short PR interval, partial right bundle branch block with left ventricular hypertrophy on voltage criteria. Over the following six years, he developed severe left ventricular hypertrophy with myocardial fibrosis (Figures 2(c) and 2(d)). In addition, his exercise tolerance worsened over two years, and he experienced “cramp-like” pain in his legs. Pediatric neurology examination confirmed reduced power, particularly distal in the upper limbs and proximally in the lower limbs. A skin and muscle biopsy showed widespread architectural abnormalities, ranging from unevenness to minicore myopathy (Table 3).

**Figure 3:**
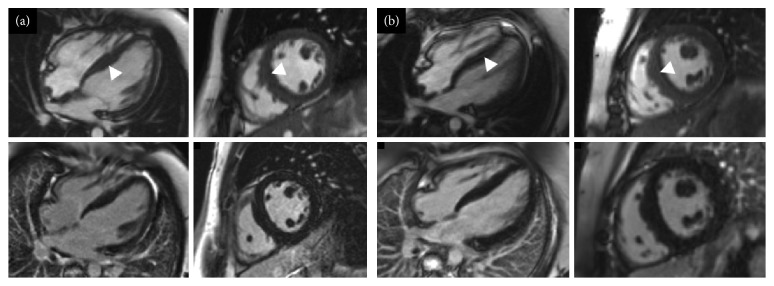
Short-axis and four chamber cine images from the initial cardiovascular magnetic resonance scans of II:2 (a) and II:3 (b) from family A. Top row for each set of four images shows cine images and the bottom row late gadolinium enhancement images. (a, b) Mild asymmetric left ventricular hypertrophy (maximum wall thickness: (a) 13 mm; (b) 14 mm). No late gadolinium enhancement is demonstrated.

II:1's younger brother (II:2 in Figure 1(a)), now 12 years old, had no previous cardiac history. At first presentation, he had good exercise tolerance; but one year later, he reported some tiredness. His initial electrocardiogram showed a short PR interval with incomplete right bundle branch block and preexcitation. Initial echocardiogram showed no evidence of an overt hypertrophic cardiomyopathy phenotype. Cardiovascular magnetic resonance imaging, 3 years later, showed early features for hypertrophic cardiomyopathy (Figure 3(a)).

II:1's younger sister (II:3 in Figure 1(a)), who is now 9 years old, reported episodic nonlocalised chest pain at age 6, worse on movement. Her first electrocardiogram showed partial right bundle branch block and voltage criteria for left ventricular hypertrophy, but the latest electrocardiography additionally showed preexcitation. The initial echocardiogram showed no phenotypic evidence of hypertrophic cardiomyopathy. However, her latest echocardiogram and cardiovascular magnetic resonance showed a mild hypertrophic cardiomyopathy phenotype (Figure 3(b)).

## 3. Family B

Family B, of South Asian origin, carries the mutation, c.1518A > C (p.Glu506Asp) (Table 1). Electrocardiographic and echocardiographic findings are listed in Table 2.

**Figure 4:**
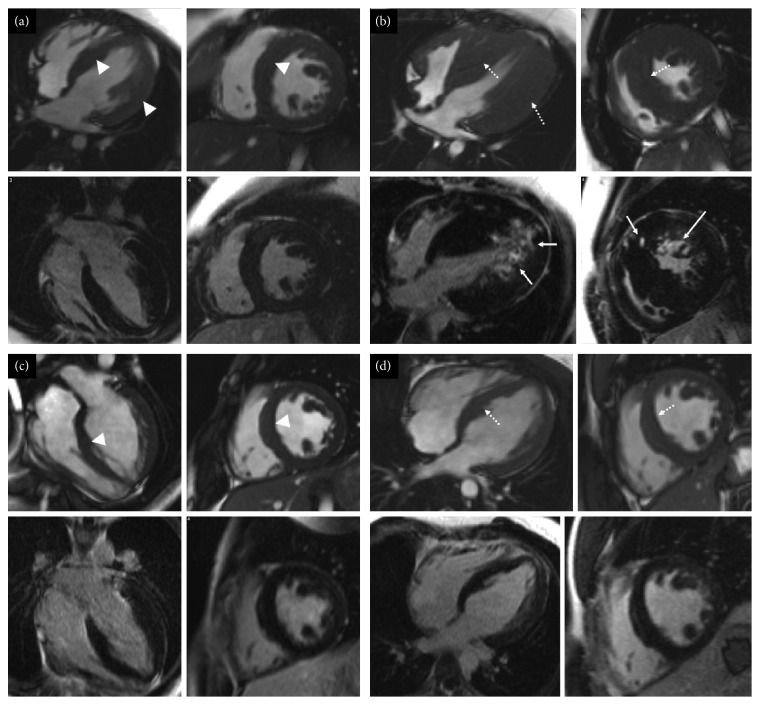
Short-axis and four chamber cine images from the initial and latest cardiovascular magnetic resonance scans of (a, b) II:1 and (c, d) II:2 from family B. Top row for each set of four images shows cine images, and the bottom row shows late gadolinium enhancement images. (a) The initial study showed asymmetric left ventricular hypertrophy (white arrowheads) with a maximum wall thickness of 15 mm, and no areas of late gadolinium enhancement were present. (b) On the latest follow-up scan, there is marked left ventricular hypertrophy (42 mm; dotted arrows) and patchy midwall v along with regions of subepicardial enhancement (white arrows). (c, d) Mild asymmetric left ventricular hypertrophy on the initial scan and the latest scan (15–17 mm). No regions of late gadolinium enhancement are present.

The proband (II:1 in Figure 1(b)) is a 16-year-old boy, diagnosed with mild concentric left ventricular hypertrophy at the age of 8. Two years later, significant concentric left ventricular hypertrophy was identified. The electrocardiogram showed signs of left ventricular hypertrophy, repolarisation abnormalities in V1 and V2, and right bundle branch block. He underwent catheter ablation of atrioventricular reentry tachycardia due to a left lateral accessory pathway at the age of 11 and was referred to the cardiomyopathy clinic, subsequent to his stepbrother's diagnosis of hypertrophic cardiomyopathy. He described increased breathlessness during low-level exercise and dizziness. Echocardiogram and cardiovascular magnetic resonance imaging showed severe hypertrophic cardiomyopathy, particularly affecting the left ventricular lateral wall and myocardial fibrosis (Figures 4(a) and 4(b)). The patient had an implantable cardioverter defibrillator implanted, due to a short PR interval and left ventricular strain electrocardiographic pattern. The chest pains he continued to experience were thought to be due to skeletal myopathy. This is when he had a diagnosis of PRKAG2 gene mutation. Skeletal muscle biopsy at age 12 years showed a few scattered small slow fibres. There was no evidence of glycogen storage nor excess acid phosphatase activity (Table 3).

The proband's maternal stepbrother (II:2 in Figure 1(b)), a 25-year-old man, was diagnosed with hypertrophic cardiomyopathy, at age 16 years. He complained about temporary episodes of chest pain associated with paraesthesia of the left arm and episodes of intermittent palpitation and confusion. The chest pain was suspected due to skeletal myopathy, but this was dismissed by the neurologist nine years later. However, no muscle biopsy was carried out. His initial electrocardiogram showed sinus rhythm with a short PR interval and voltage criteria for left ventricular hypertrophy, with inferolateral repolarisation abnormality. Echocardiogram and cardiovascular magnetic resonance demonstrated mild asymmetrical hypertrophy of the basal and midseptum with a maximum thickness of 15mm and inducible myocardial ischaemia, but with no fibrosis (Figures 4(c) and 4(d)).

**Figure 5:**
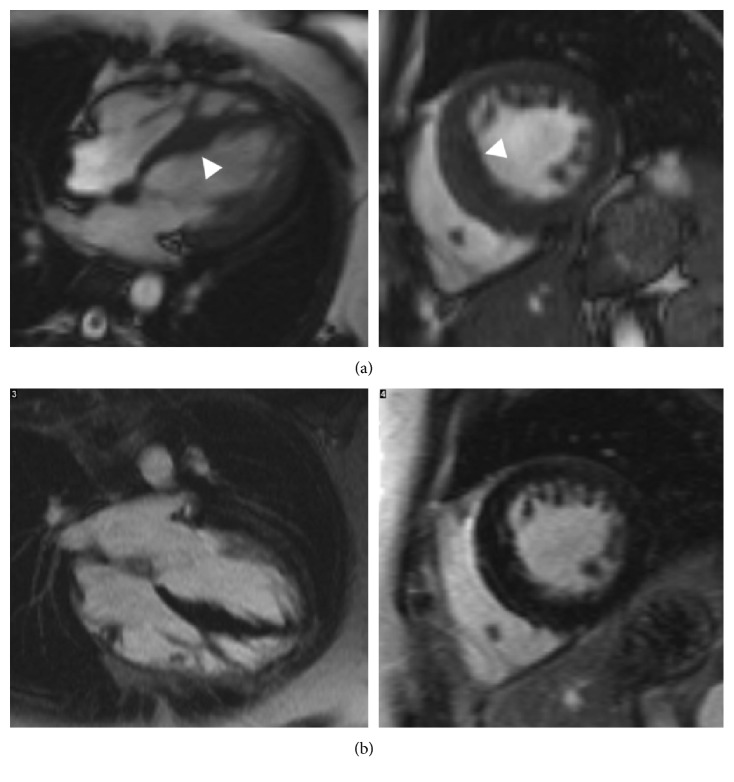
Initial cardiovascular magnetic resonance scan of patient I:2 from family B taken in 2009. (a) Cine images and (b) late gadolinium enhancement images. Asymmetric left ventricular hypertrophy (15 mm), no areas of late gadolinium enhancement.

The proband's mother (I:2 in Figure 1(b)), a 52-year-old woman, presented at age 44, with occasional transient palpitations, dizzy spells, and mild tinnitus. She was diagnosed as having asymmetric left ventricular septal hypertrophy. Her electrocardiogram at presentation showed sinus bradycardia, and T-wave inversion in the precordial and inferior leads with deep S waves inferiorly in V4-6. Later, electrocardiograms showed a short PR interval with intermittent delta waves and left ventricular hypertrophy. Initial echocardiogram and cardiovascular magnetic resonance showed asymmetric septal hypertrophy with a maximum thickness of 15 mm, with no left ventricular outflow tract obstruction and no fibrosis (Figure 5). Chest pain was thought to be due to musculoskeletal pain. A dual-chamber implantable cardioverter defibrillator was implanted for symptomatic bradycardia, but she later required cardiac transplantation. The patient now requires a walking stick for mobility.

## 4. Discussion

PRKAG2 is a fascinating cause of hypertrophic cardiomyopathy and skeletal myopathy. It mimics sarcomeric protein mutation but has differing and often more systemic features that present a distinct management challenge.

The PRKAG2 gene encodes the γ2 isoform of the gamma subunit of the AMP-activated protein kinase enzyme [5]. This enzyme senses and responds to energy demands within mammalian cells and is activated in different tissues, including cardiac and skeletal muscles. Activation of the enzyme is triggered by adenosine monophosphate binding to the γ2 subunit and mutations in this subunit, leading to defects in glycogen-dependent pathways, causing glycogen accumulation and exercise-induced skeletal muscular symptoms [4]. Histopathology shows excess intracellular glycogen deposition in vacuoles, myocyte enlargement with no disarray and in advanced stages, extracellular fibrosis [2].

In PRKAG2-type hypertrophic cardiomyopathy, the degree of hypertrophy depends on the extent of myocyte enlargement, secondary to glycogen accumulation. Left ventricular hypertrophy occurs more commonly in a concentric pattern; however, a recent cardiovascular magnetic resonance study showed that eccentric left ventricular hypertrophy can also be seen with PRKAG2 mutations, with early syndromes exhibiting a focal midinferolateral pattern of left ventricular hypertrophy, with advanced stages presenting with a more diffused pattern, focusing in the interventricular septum [2]. Unfortunately, it is unclear why some patients present with severe hypertrophic cardiomyopathy and other patients show no left ventricular hypertrophy [6]. PRKAG2 is, however, commonly misdiagnosed as sarcomeric hypertrophic cardiomyopathy, and so, it is important to note that left ventricular hypertrophy in sarcomeric hypertrophic cardiomyopathy is typically asymmetric and predominantly involves the basal interventricular septum, the lateral wall, posterior septum, and left ventricular apex [2]. PRKAG2 mutations that affect the skeletal muscles change single amino acids in the γ2 subunit of AMP-activated protein kinase enzyme causing myocardial dysfunction and myopathy due to depletion of adenosine triphosphate supplies. These patients experience muscle pain and stiffness, especially after exercise [5].

Compared to others, our report highlights the variability of clinical and cardiac imaging findings in adult and also pediatric patients. Gollob et al. showed that the onset in childhood and adolescence is more severe, with clinical presentation of symptomatic arrhythmias occurring in early childhood, but without left ventricular hypertrophy [3]. This is partly different to our findings, as children from both families presented with early onset hypertrophic cardiomyopathy with a rapid progression in some (*n* = 2 out of 5) children (Figures 2(c), 2(d), 4(a), and 4(b)).

Different mutations in the PRKAG2 gene can present in a similar manner, but with a few variants [3]. By studying two families with the PRKAG2 mutation c.1518A > C (p.Glu506Asp) and c.1463A > T (p.Asn488lle), we can see that children typically presented first with electrocardiographic abnormalities, with no hypertrophic cardiomyopathy detected on imaging until more advanced stages of the disease, whereas adults presented with hypertrophic cardiomyopathy first.

Our findings also show that that the onset and course of the disease can be different between family members with the same mutation. This absence or variable expression of hypertrophy could be due to the specific mutation Arg531Gly, as seen in family A, where the children first showed evidence of preexcitation with a short PR interval and later developed signs of hypertrophic cardiomyopathy [7]. However, their father was identified to have hypertrophic cardiomyopathy on imaging at his first appointment. Aggarwal et al. also emphasised the common diagnostic scenario of ongoing arrhythmic problems in patients with hypertrophic cardiomyopathy and/or Wolff–Parkinson–White syndrome, despite ablation of the suspected accessory pathways. This was seen in our proband from family B, who underwent ablation of his accessory pathway but still showed electrophysiological abnormalities [6].

In patients with c.1463A > T, skeletal myopathy is found in 15% and from family B, we report the same in the c.1518A > C variant: a new finding [1]. It is not understood why there is a marked discrepancy between the severity of cardiac and skeletal muscle symptoms which may be due to more abundance of the AMP-activated protein kinase enzyme γ2 subunit in the heart [3].

We have shown that the specific pathogenomic mutations, c.1518A > C (p.Glu506Asp) and c.1463A > T (p.Asn488lle), cause HCM and skeletal myopathy. There should be a low index of suspicion for PRKAG2 mutations, where there is a combination of chest pain, reduced exercise tolerance, short PR interval and possible bundle branch block even before concentric left ventricular hypertrophy has developed, and clinicians should be alerted once worsening electrocardiogram and signs of fatigue are identified. Currently, early diagnosis does not appear to change management, as there is no targeted therapy for the various mutations of AMP-activated protein kinase enzyme, but it may improve management of the symptoms and outcome. Nevertheless, early diagnosis does allow an explanation of the various systemic symptoms which may lead to extrasupport for daily activities and assistance in school or work and which may help to alleviate the psychological impact of this syndrome.

We have described the clinical and investigative features of PRKAG2 mutation in two families. We have demonstrated that the PRKAG2 mutations c.1518A > C (p.Glu506Asp) and c.1463A > T (p.Asn488lle) present with skeletal myopathy and preexcitation at an early stage, and in some cases before significant cardiac hypertrophy has developed. There should be a low index of suspicion and greater awareness of this condition in patients with systemic symptoms, electrocardiographic changes, and features of hypertrophic cardiomyopathy in children, adolescents, and adults. Targeted treatments are still awaited.

## Figures and Tables

**Table 1:** Anthropometric characteristics.

Demographics	Family A				Family B		
	I:1	II:1	II:2	II:3	I:2	II:1	II:2
Sex	M	M	M	F	F	M	M
Age (initial presentation, y)	33	11	7	5	44	8	16
Height (cm)	176	140	131	123	160	156	175
Weight (kg) (2.s.f)	71	36	32	30	64	49	57

**Table 2:** Electrocardiographic, echocardiographic, and biomarker findings.

Features at examinations	Family A				Family B		
	I:1	II:1	II:2	II:3	I:2	II:1	II:2
Latest ECG							
LV hypertrophy	+	+	+	+	+ (initial ECG)	+ (initial ECG)	+
Preexcitation	+	+	+	+	+ (initial ECG)	+ (initial ECG)	+
WPW	+	+	+	+	+ (initial ECG)	− (initial ECG)	+
Pacemaker/ICD					+	+	

Initial echocardiography							
EF (%)	60–65	61	—	50	76.8	75	73.4
FS (%)	—	42		20.5	36.4	44	35.7
LVEDD (cm)	4.72	3.7	—	3.57	3.9	—	4.2
LVPWd (cm) (±SD)	1.05	1.1 (+3.9)	—	0.87 (+2.1)	1.27	—	1.5
IVSD (cm) (±SD)	1.31	1.1 (+3.2)	—	0.55 (−1.7)	1.43	—	1.7

Latest echocardiography							
EF (%)	67	65	60	57	61	66	65
FS (%)	—	58	45	39	35.2	37.9	—
LVEDD (cm)	4.91	3.64	4.64	4.16	4.04	3.91	4.71
LVPWd (cm) (±SD)	0.98	1.74 (+6.6)	0.82 (−0.3)	1.0 (+1.5)	1.18	2.02 (+8.1)	1.26
IVSD (cm) (±SD)	1.16	1.7 (+4.8)	0.82 (−0.6)	1.4 (+3.9)	1.47	2.52 (+12.7)	1.4

Biomarker (initially)							
Troponin (ng/L)	<40	<40	<40	<20	90	4200	40
Creatine kinase (U/L)	131	68	286	103	98	350	125
BNP (ng/L)	21	28	14	22	253	16	<42

Biomarker (latest)							
Troponin (ng/L)	—	83	<20	<20	351	528	26
Creatine kinase (U/L)	—	76	115	81	71	156	147
BNP (ng/L)	—	234	7	11	55	84	11

**Table 3:** Muscle biopsy results.

Muscle biopsy features	Family A	Family B
	II:1	II:1
Source of sample	Skeletal muscle, unspecified site.	Right vastus lateralis
Findings	Myopathy with minicores. Minimal fibre size variation. Main abnormality is the widespread architectural abnormality in fibres seen in the oxidative stains, ranging from unevenness to minicores and present in both fibre types.	Shows minimum departure from the normal with presence of a few scattered small slow fibres.
